# Post-extubation laryngitis in children: diagnosis, management and follow-up

**DOI:** 10.1016/j.bjorl.2024.101440

**Published:** 2024-05-02

**Authors:** Elaine Costa, Débora Bressan Pazinatto, Luciahelena Prata Trevisan, Rebecca Maunsell

**Affiliations:** Disciplina de Otorrinolaringologia Cabeça e Pescoço, Faculdade de Ciências Médicas, Universidade Estadual de Campinas (UNICAMP), Brazil

**Keywords:** Laryngitis, Extubation, Children, Pediatric intensive care units

## Abstract

**Objectives:**

To describe the occurrence of post-extubation laryngitis, analyze its one-year evolution, and correlate laryngeal lesions with clinical outcomes.

**Methods:**

Retrospective study including children up to 13 years old at a tertiary hospital between March 2020 and March 2022 with diagnosis of post-extubation laryngitis confirmed by endoscopic examination. Exclusion criteria were prior history of intubation or anatomical airway abnormalities. Medical records were reviewed to characterize patients, underlying diagnosis, laryngeal lesions, treatment, and outcomes at 12-month follow-up.

**Results:**

The study included 38 endoscopically confirmed post-extubation laryngitis cases, corresponding to 86.4% of suspected cases. The mean age was 13.24 months, and 60.5% were male. Acute respiratory failure was the leading cause of intubation. Initial treatment was clinical, and initial diagnosis was defined by nasopharynoglaryngoscopy and/or Microlaryngoscopy and Bronchoscopy (MLB) findings. Initial diagnostic MLB was performed in 65.7% of the patients. Approximately half (53%) of the patients exhibited moderate or severe laryngeal lesions. When compared to mild cases, these patients experienced a higher rate of extubation failures (mean of 1.95 vs. 0.72, *p* = 0.0013), underwent more endoscopic procedures, and faced worse outcomes, such as the increased need for tracheostomy (*p* = 0.0001) and the development of laryngeal stenosis (*p* = 0.0450). Tracheostomy was performed in 14 (36.8%) children. Patients undergoing tracheostomy presented more extubation failures and longer intubation periods. Eight (21%) developed laryngeal stenosis, and 17 (58.6%) had complete resolution on follow-up.

**Conclusion:**

Post-extubation laryngitis is a frequent diagnosis among patients with clinical symptoms or failed extubation. The severity of laryngeal lesions was linked to a less favorable prognosis observed at one-year follow-up. Otolaryngological evaluation, follow-up protocols, and increased access to therapeutic resources are essential to manage these children properly.

**Level of evidence:**

Level 4.

## Highlights

• Post-extubation laryngitis was confirmed in the majority of suspected cases.
• Bedside nasopharyngolaryngoscopy assisted, but over half required microlaryngoscopy.
• Laryngeal stenosis is associated with lesion severity and extubation failures.
• Close follow-up after discharge prevented any additional tracheostomies.
• Prognosis is influenced by comorbidities, lesion severity, and tracheostomy.

## Introduction

Post-Extubation Laryngitis (PEL), or post-intubation laryngitis, consists of an inflammation of the larynx caused by an endotracheal tube and noticeably diagnosed after its removal. It’s a potentially severe complication of endotracheal intubation with variable incidence, between 2.4% and 40%, depending on the studied population and criteria established.^1^, ^2^, ^3^, ^4^ Damage to the columnar pseudostratified epithelium begins soon after intubation and can result in injuries of varying severity.^5^ Severe cases can culminate in extubation failures, in which reintubation increases morbidity and mortality.^6^

Survival rates in Pediatric Intensive Care Units (PICUs) have increased dramatically in recent decades and the advance of pediatric airway management seems to be a contributing factor. Endotracheal intubation is widely used in increasingly younger, premature, and low birth weight patients.^7^ While intubation is necessary, it may lead to temporary or permanent airway damage, such as laryngeal edema, granulation tissue formation, vocal fold paralysis, Subglottic Stenosis (SGS), glottic or tracheal stenosis.^8^

In 1969, Lindholm correlated endotracheal intubation with laryngeal and tracheal injuries, proposing that the inadequate size of the endotracheal tube was the main factor for developing subsequent glottic lesions.^9^, ^10^ Studies indicate that the main risk factors include intubation lasting over 24 h, an inappropriate size of the endotracheal tube, traumatic intubation often performed by unqualified professionals or those in training, the need for reintubation or tube exchange, and concurrent respiratory tract infections.^7^, ^11^

Post-extubation laryngitis impacts respiratory outcomes, which can range from a self-limited condition without sequelae to severe airway involvement leading to morbidities. The need for a tracheostomy potentially impairs an individual's quality of life and may lead to rehospitalizations.^7^

Upon clinical suspicion, the diagnosis requires endoscopic confirmation. Flexible bedside Nasopharyngolaryngoscopy (NPL) is the initial exam due to low cost and risk and good diagnostic accuracy.^12^ Microlaryngoscopy and Bronchoscopy (MLB) under general anesthesia can also be performed with a more accurate evaluation of the posterior glottis, subglottis and trachea, allowing for therapeutic procedures simultaneously.^13^

Exam findings can be analyzed using internationally validated classifications to define the degree of airway involvement and estimate the prognosis and respiratory outcome.^8^, ^9^, ^13^, ^14^

Understanding these patients' epidemiological conditions and clinical variables contributes to more effective strategies to prevent and treat acquired laryngeal lesions.^2^, ^7^, ^10^, ^11^^,^^15^

A care flow of patients by the pediatric otolaryngology team within the PICU at the Hospital de Clínicas affiliated with the Universidade Estadual de Campinas (HC Unicamp) has been established in the past seven years. Since early 2020, all attended cases have been documented and recorded in a specific spreadsheet with data relating to patients' characteristics, underlying pathologies, endoscopic findings, and treatments instituted.

This study aims to describe the occurrence of endoscopically confirmed PEL in children who presented symptoms after extubation and/or extubation failure in a tertiary public university hospital, analyze its one-year evolution, and correlate laryngeal lesions with clinical outcomes.

## Methods

### Research design

Retrospective study.

### Sampling

Interconsultation requests from the PICU to the pediatric otorhinolaryngology team at a tertiary university hospital were reviewed from March 2020 to March 2022. Requests due to extubation failure or symptoms of stridor and dysphonia after extubation were selected for further analysis.

The pediatric otolaryngology team at the Hospital de Clínicas of Unicamp, which is a teaching hospital, has provided care for patients within the PICU. Since early 2020, we have documented patients’ pathologies, endoscopic findings, and treatments in a spreadsheet.

Patients with confirmed PEL through NPL or MLB performed by otolaryngology were included. Ages ranged from 30 days to 13 years, 11 months, and 29 days, corresponding to the admission age range at the PICU. Children with prior intubation history, anatomical airway malformations, or diagnosed laryngeal pathologies were excluded.

At HC Unicamp, the pediatric otolaryngology airway team is responsible for all airway assessments and tracheotomies in children. The team consists of two trained otolaryngologists, a thoracic surgeon, and training pediatric otolaryngology fellows. Since 2016, when PEL is clinically suspected, the intensive care team has implemented standardized therapeutic protocols as defined jointly with the pediatric otolaryngology team.^16^, ^17^ MLB was conducted upon lack of symptom improvement after 72 h post-extubation with clinical treatment, two failed extubations, or if NPL could not rule out severe lesions.^16^, ^17^

Extubated PICU patients underwent bedside nasopharyngolaryngoscopy without additional sedation. A 3.2 mm flexible fiber coupled to a smartphone with an adaptor, camera app, and portable light source (M-scope, GBEF Telefonia, São Paulo, Brazil) was used.

The institution’s Research Ethics Committee approved this project under number CAAE 48736621.8.0000.5404.

### Data collection

Medical records of all patients who attended the inclusion criteria were reviewed to collect data on variables of interest during hospital admission and a 12-month follow-up period.

Data collected included: sex, age at the time of assessment, comorbidities, reason for intubation, duration until the first elective extubation attempt, total time with endotracheal tubes, total days with invasive mechanical ventilation, length of stay in the PICU and hospital, number of extubation failures, and the count of NPL and MLB performed.

Laryngeal injuries were categorized based on NPL and/or MLB findings according to the CALI classification as mild, moderate, or severe.^8^ The definition of comorbidity considered the following categories: genetic syndromes, neuropathy, heart disease, gastrointestinal disorders, pneumopathy, and nephropathy.^18^

Outcomes at the end of 12 months of follow-up were considered in two categories: Resolved cases, which were children with no tracheostomy and no respiratory symptoms, and unresolved cases, which included either those still with a tracheostomy (waiting for diagnostic procedures, compensation of underlying conditions or reconstructive surgeries) or not with a tracheostomy but with residual symptoms that required further care or follow up.

## Results

**Figure 1:**
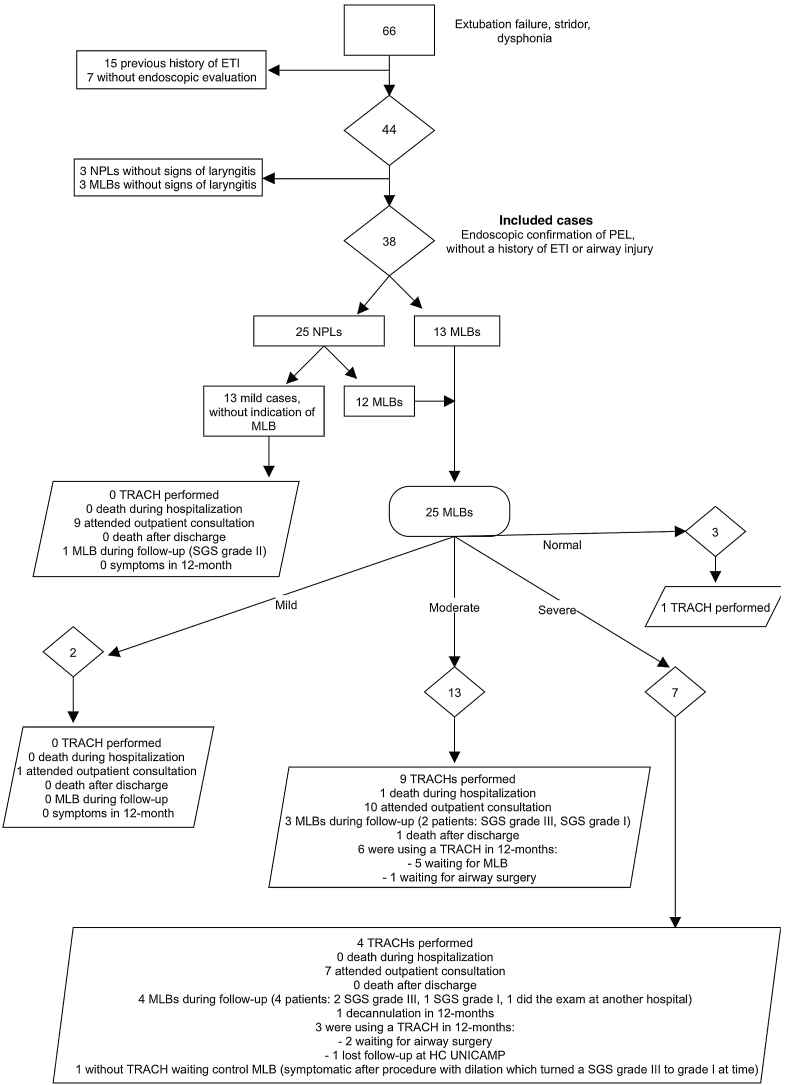
Summary of case selection, endoscopic evaluation performed, severity classification and need for tracheostomy. *Three patients classified as mild PEL by NPL showed normal MLB. ETI, Endotracheal Intubation; MLB, Microlaryngoscopy and Bronchoscopy; TRACH, Tracheostomy; SGS, Subglottic Stenosis; NPL, Nasopharyngolaryngoscopy; DX, Diagnosis; EVAL, Evaluation; HC, Hospital de Clínicas; PEL, Post-Extubation Laryngitis.

This study included 38 endoscopically confirmed PEL cases (NPL and/or MLB) over 2 years. Sixty-six requests for otolaryngology evaluation in children with post-extubation stridor, dysphonia, and/or extubation failure were reviewed. Fifteen had previous intubation history, and seven lacked endoscopic assessment (Fig. 1). Among 44 with suspected PEL who underwent NPL/MLB without prior intubation, 38 were confirmed PEL and included for analysis.

Acute respiratory failure was the primary indication for intubation in 68.4% of cases, followed by neurological causes (23.7%) and intubation for procedure/surgery/transport (7.9%). Males were majority (60.5%), and comorbidities were present in 42.1% of cases.

The mean age of the patients was 13.24 months (median = 6, Standard Deviation [SD = 23.27]), with ages ranging from 1 to 115 months. The mean duration until the first extubation was 6.32 days (median = 5, SD = 8.22), ranging from 0 to 47 days. Patients remained intubated for a mean total duration of 18.68 days (median = 12, SD = 18.54), ranging from 1 to 84 days. The mean number of extubation failures was 1.37 (median = 1, SD = 1.17), ranging from 0 to 4 failures. Finally, the mean length of hospital stay was 57.71 days (median = 35, SD = 90.82), ranging from 8 to 565 days. Overall, while average ventilation durations and hospital stays were under three weeks, there was wide variability across patients, with some remaining intubated for up to 12 weeks and hospitalized for over 1.5 years.

Among clinically suspected cases, the occurrence of confirmed post-extubation laryngitis was 86.4% (38/44). Before evaluation by the otolaryngology airway team, clinical interventions by the PICU staff included: proton pump inhibitors, intravenous corticosteroids, inhaled epinephrine, and/or non-invasive ventilation as indicated.

### Diagnostic and therapeutic procedures

NPL was the initial evaluation for 25 patients, among whom 13 showed mild laryngitis, which resolved with clinical measures without needing MLB under general anesthesia, and 12 required subsequent MLB. MLB was the initial procedure for 13 reintubated patients. Therefore, MLB was performed for initial diagnostic and/or therapeutic purposes in 65.79% (25/38) of PEL cases.

Over the two years, a total of 35 MLBs were performed during hospitalization under general anesthesia to evaluate children with PEL. Another nine endoscopic procedures were performed during outpatient follow-up. On average, each patient underwent 1.16 MLBs (median = 1, SD = 1.17, range = 0–5).

Endoscopic treatment through MLB included one or more of the following: removal of fibrinous tissue, intralesional corticosteroid injection, dilation with a balloon, removal of potentially obstructive granulation tissue, local application of ointment (Regencel®), and/or tracheostomy. Characteristics of laryngeal lesions dictated decision-making.

### Severity of laryngeal lesions

All cases were classified according to severity based on endoscopic evaluation with NPL and/or MLB. Distribution of acute laryngeal lesions due to PEL were: 19% severe, 34% moderate, and 47% mild cases following the CALI classification.

There were three cases with no abnormal findings on MLB that had been previously classified as mild based on NPL findings (Fig. 1). These cases were probably resolved in the time transcurred from NPL and MLB.

Table 1 compares the mild severity group (n = 18) and the moderate or severe groups (n = 20). Patients with mild laryngitis had significantly less need for a tracheostomy (1 vs. 13 procedures, respectively, *p* = 0.0001, Chi-Square test). Patients with moderate or severe laryngitis had a significantly higher average of extubation failures than mild cases (1.95 ± 1.19 and 0.72 ± 0.75, respectively, with *p* = 0.0013, Mann-Whitney test).

**Table 1:** Comparison between CALI severity groups regarding number of MLB's, presence of laryngeal stenosis, resolution on follow-up, length of intubation, mechanical ventilation and total time in PICU.

Variable	CALI mild (n = 18)	CALI moderate or severe (n = 20)	*p*-value
Total MLBs			**<0.0001**^b^
**0**	12 (66.7%)	0 (0.0%)	
1	6 (33.3%)	10 (50.0%)	
>1	0 (0.0%)	10 (50.0%)	
Laryngeal stenosis			**0.0450**^c^
Yes	1 (5.6%)	7 (35.0%)	
No	17 (94.4%)	13 (65.0%)	
Resolved cases (without TRACH/respiratory symptoms)			**0.0032**^c^
Yes	11 (91.7%)	6 (35.3%)	
No	1 (8.3%)	11 (64.7%)	
Total time in ETI (days)			**<0.0001**^a^
Mean ± SD	7.39 ± 5.04	28.85 ± 20.43	
Median (min‒max)	5.50 (2.00‒18.00)	25.50 (1.00‒84.00)	
Time in IMV (days)			**<0.0001**^a^
Mean ± SD	7.39 ± 5.04	59.20 ± 118.57	
Median (min‒max)	5.50 (2.00‒18.00)	30.00 (1.00‒554.00)	
Time in PICU (days)			**<0.0001**^a^
Mean ± SD	15.22 ± 10.93	54.85 ± 32.97	
Median (min‒max)	12.00 (3.00‒40.00)	48.50 (9.00‒124.00)	

CALI, Classification of Acute Larynx Injuries; TRACH, Tracheostomy; SD, Standard Deviation; ETI, Endotracheal Intubation; IMV, Invasive Mechanical Ventilation; PICU, Pediatric Intensive Care Unit; MLB, Microlaryngoscopy and Bronchoscopy.

a Based on the Mann-Whitney test.

b Based on the Chi-square test.

c Based on Fisher’s exact test.

There was also a statistically significant difference in the number of MLBs performed during hospitalization and follow-up, with moderate or severe cases requiring more procedures.

Increased severity of lesions was associated with a higher need for tracheostomy (*p* = 0.0001), more significant development of laryngeal stenosis (*p* = 0.0450), lower resolution within a 12-month follow-up (*p* = 0.0032), as well as longer durations of endotracheal tube use, need for invasive mechanical ventilation, and ICU admission (*p* < 0.0001 for all variables). CALI severity groups showed no differences regarding the presence of comorbidities (*p* = 0.0897) and sex (*p* = 0.5520), based on Chi-Square test.

### Tracheostomy

At the end of the hospitalization period, 14 patients (36.84%) had undergone tracheostomy due to unsuccessful extubation attempts despite treatment of PEL and/or the need for continuous ventilatory support.

Analysis of the association between tracheostomy and endoscopic procedures, we observed that an escalation of one MLB during hospitalization was associated with a 21-fold increase in the likelihood of tracheostomy (Table 2).

**Table 2:** Simple and multiple logistic regressions for factors associated with performing a tracheostomy (modeling probability of “yes”).

	Simple analysis			
Variable	Categories	*p-*value	OR	95% CI
Comorbidities	Yes vs. No	0.0077	7.500	1.703; 33.033
Extubation failures	–	0.0059	3.071	1.381; 6.828
MLBs during hospitalization	–	0.0035	21.048	2.721; 162.836
CALI	Moderate or Severe vs. Mild	0.0023	31.571	3.442; 289.597

	Multiple analysis (stepwise variable selection process)			
Variable		*p*-value	OC	95% CI
MLBs during hospitalization		0.0035	21.048	2.721; 162.836

OR, Odds Ratio; 95% CI, 95% Confidence Interval for OR; TRACH, Tracheostomy; CALI, Classification of Acute Larynx Injuries; MLB, Microlaryngoscopy and Bronchoscopy.

Table 3 compares the tracheostomy group vs. non-tracheostomy. Patients with comorbidities had higher tracheostomy frequency (62.5% [10/16] vs. 18.2% [4/22] without comorbidities, *p* = 0.0052, Chi-Square test).

**Table 3:** Comparison between presence of TRACH vs. absence of TRACH.

Variable	Trach	Trach	*p*-value
	Yes (n = 14)	No (n = 24)	
Total MLBs			**<0.0001**^b^
0	0 (0.0%)	12 (50.0%)	
1	5 (35.7%)	11 (45.8%)	
>1	9 (64.3%)	1 (4.2%)	
Resolved cases (without TRACH/respiratory symptoms)			**<0.0001**^b^
Yes	2 (15.4%)	15 (93.8%)	
No	11 (84.6%)	1 (6.3%)	
Extubation failures			**0.0012**^a^
Mean ± SD	2.14 ± 1.03	0.92 ± 1.02	
Median (min-max)	2.00 (0.00‒4.00)	1.00 (0.00‒4.00)	

TRACH, Tracheostomy; ETI, Endotracheal Intubation; SD, Standard Deviation; IMV, Invasive Mechanical Ventilation; PICU, Pediatric Intensive Care Unit; MLB, Microlaryngoscopy and Bronchoscopy.

a Based on the Mann-Whitney test.

b Based on Fisher’s exact test.

The tracheostomy group had longer hospital stay (mean ± SD [min‒max] 111.29 ± 133.69 [36–565] vs. 26.46 ± 19.99 [8–88] days, *p* < 0.0001, Mann-Whitney test); longer PICU stay (65 ± 32.66 [23–124] vs. 19.21 ± 14.96 [3–59] days, *p* < 0.0001, Mann-Whitney test); and prolonged intubation (32.64 ± 20.91 (2–84) vs. 10.54 ± 10.88 (1–52) days, *p* < 0.0002, Mann-Whitney test).

### Laryngeal stenosis

Laryngeal stenosis occurred in 21% (8/38) of the sample, with 7 subglottic and 1 posterior glottic stenosis after 12 months. Subglottic cases: 3 grade I, 1 grade II, 3 grade III.

After 12 months, some awaited MLB for chronic lesion diagnosis, potentially underestimating stenosis cases. One mild PEL case developed subglottic stenosis 15 days after initial evaluation, this was diagnoses through MLB after discharge, and successfully treated with dilatation remaining asymptomatic on follow-up.

As can be appreciated in Table 4, there was no statistically significant association between comorbidities and the development of laryngeal stenosis (*p* = 0.6984).

**Table 4:** Comparison between laryngeal stenosis development vs. no stenosis.

Variable	Laryngeal Stenosis	Laryngeal Stenosis	*p*-value
	Yes (n = 8)	No (n = 30)	
Comorbidities			0.6984^b^
Yes	4 (50.0%)	12 (40.0%)	
No	4 (50.0%)	18 (60.0%)	
MLBs during hospitalization			**0.0218**^b^
0	1 (12.5%)	12 (40.0%)	
1	2 (25.0%)	14 (46.7%)	
>1	5 (62.5%)	4 (13.3%)	
CALI			**0.0450**^b^
Mild	1 (12.5%)	17 (56.7%)	
Moderate or Severe	7 (87.5%)	13 (43.3%)	
ETI duration until 1st extubation (days)			**0.0418**^a^
Mean ± SD	9.00 ± 7.13	5.60 ± 8.45	
Median (min‒max)	7.50 (1.00‒25.00)	3.00 (0.00‒47.00)	
Extubation failures			**0.0473**^a^
Mean ± SD	2.13 ± 1.25	1.17 ± 1.09	
Median (min‒max)	2.00 (0.00‒4.00)	1.00 (0.00‒4.00)	

CALI, Classification of Acute Larynx Injuries; ETI, Endotracheal Intubation; SD, Standard Deviation; MLB, Microlaryngoscopy and Bronchoscopy.

a Based on the Mann-Whitney test.

b Based on Fisher’s exact test.

### Outcomes

After hospital discharge, 76.32% of participants (29 out of 38) attended outpatient consultations, and nine were lost to follow-up. At the end of the 12 months, 17 of the 29 patients (58.62%) were considered resolved cases; amongst these two that had undergone tracheostomy had achieved decannulation. After hospital discharge, no child required a tracheostomy.

Of 12 unsolved cases: 3 had tracheostomy and laryngeal stenosis awaiting reconstructive surgery, 8 had tracheostomy awaiting airway staging, and 1 without tracheostomy had grade I subglottic stenosis with recurrent respiratory episodes needing further endoscopy.

Factors associated with the resolution of PEL included (Table 5): mild cases (*p* = 0.0032, Fisher’s exact test), and the absence of tracheostomy (*p* < 0.0001, Chi-Square test). There was no statistically significant association with the presence of laryngeal stenosis (*p* = 0.2180, Fisher’s exact test). Additionally, 88.2% of patients with PEL resolution at follow-up had no comorbidities, while 33.3% of patients without PEL resolution had no comorbidities (*p* = 0.0045, Fisher’s exact test).

**Table 5:** Comparison between resolution vs. no resolution at the end of post-discharge follow-up.

Variable	Resolution	Resolution	*p*-value
	Yes (n = 17)	No (n = 12)	
CALI			**0.0032**^c^
Mild	11 (64.7%)	1 (8.3%)	
Moderate or Severe	6 (35.3%)	11 (91.7%)	
TRACH			**<0.0001**^b^
Yes	2 (11.8%)	11 (91.7%)	
No	15 (88.2%)	1 (8.3%)	
Outpatient consultations			**0.0008**^a^
Mean ± SD	2.29 ± 1.21	3.92 ± 1.08	
Median (min–max)	2.00 (1.00‒5.00)	3.50 (3.00‒6.00)	

CALI, Classification of Acute Larynx Injuries; TRACH, Tracheostomy; SD, Standard Deviation.

a Based on the Mann-Whitney test.

b Based on the Chi-Square test.

c Based on Fisher’s exact test.

When analyzing factors associated with case resolution through multiple logistic regression, the presence of a tracheostomy remained the only factor in the final model. Therefore, the absence of tracheostomy was associated with an approximately 82 times increase in the chance of resolution during the analyzed period (*p* = 0.0006; OR = 82.475; 95% CI 6.615; - Not estimated).

During hospitalization, one death unrelated to PEL occurred due to a severe neurological condition. In follow-up, one tracheostomized child died from cardiac decompensation at 12 months while awaiting MLB, counted as unresolved PEL.

## Discussion

The present study revealed a high occurrence (86.4%) of confirmed PEL among PICU patients with clinical suspicion. Although respiratory symptoms are paramount in practice to raise suspicion, endoscopic confirmation is more specific, particularly in moderate to severe cases, as other authors show.^19^ Proper management of these cases under MLB can mitigate morbidity from acute and chronic laryngeal injuries after extubation.^9^, ^20^, ^21^, ^22^, ^23^ Also, continuous follow-up has proven essential, as seen by the need for further MLBs and even treatment of ongoing laryngeal stenosis in cases of PEL, even those considered mild, as described in one of the described cases.

Acute respiratory failure accounted for 68.4% (n = 26) of cases as the primary cause of orotracheal intubation. Jorgensen et al. reported a 40% prevalence of airway complications after extubation in children with acute bronchiolitis.^2^ As a common PICU admission reason requiring invasive ventilation, acute respiratory failure results from lung parenchyma diseases, airway obstruction, or neuromuscular dysfunction.^24^ Respiratory tract infection during intubation may raise the risk for PEL.^11^ Careful endotracheal intubation and extubation of children with known airway inflammation may reduce PEL.^12^, ^25^

Patients with comorbidities exhibited more severe laryngeal injuries after extubation, higher tracheostomy need, and lower 12-month resolution compared to previously healthy individuals. Comorbidities are particularly relevant when it comes to airway healing and inflammation. Experienced airway surgeons have recognized this and have even been included in a modified classification of laryngeal stenosis, given its impact on surgical outcomes and prognosis.^26^

Although nasopharyngolaryngoscopy in the PICU may lack accuracy, needing additional MLBs,^13^ in the current study, NPL sufficiently served as the initial examination, avoiding diagnostic laryngoscopy in 13 (34.21%) mild cases. Otolaryngologists working in the PICU should be trained to perform these first-line examinations and indicate the need for further MLB, depending on the progression of symptoms or inconclusive findings on NPL.

Brazil has 51 university hospitals but only nine pediatric otolaryngology specialty training programs. Those are located in the country's southern, southeastern, and central-western regions and offer 1–3 positions per academic year.^27^, ^28^ Airway management experience is still critical, particularly knowledge of other contributing factors to airway dynamics and airway healing in small children. Identifying and treating acute laryngeal lesions due to PEL and tracheostomy indications differ enormously from those for adult patients. This should be addressed in the training of pediatric otolaryngologists.^29^

Over half (65.79%) of patients developing PEL required at least one MLB. This substantial demand justifies the reformulation of logistics to establish dedicated specialized airway teams and operating room availability for these patients.

Laryngeal injury severity correlates with adverse outcomes in post-extubation laryngitis.^8^ Our findings align with the literature, showing moderate/severe cases are associated with more tracheostomies, endoscopic procedures, extubation failures, and subglottic stenosis. This is helpful information when addressing parental and PICU staff expectations.

Laryngeal stenosis occurred in 21% of cases with PEL. A prior study showed a 29.8% stenosis rate in patients with laryngeal findings after intubation.^30^ Other descriptions of these rates typically include all admissions or all intubated patients, not discriminating the cases with PEL.

The high tracheostomy rate (36.84%) most probably reflects the degree of laryngeal injury and the patient’s general condition severity. Although tracheostomies were associated with worse outcomes, these patients were also the most severe cases of PEL with the most comorbidities and pre-tracheostomy procedures; this data may be biased.

Prolonged hospitalization increases healthcare costs, making managing this condition essential for cost reduction.^31^, ^32^ Determining if earlier tracheostomies could abbreviate hospitalizations remains unanswered, given the multifactorial nature of indication of ventilatory support needs and development of acute laryngeal lesions. Nevertheless, tracheostomies also represent additional airway damage and morbidity.^33^, ^34^ Thus, our airway team advocates airway evaluation and case-to-case discussion before deciding whether to perform a tracheostomy.

A multidisciplinary approach is essential in PEL management, as endorsed by a recent expert-bases opinion on PEL diagnosis/treatment in Brazil.^17^ However, many PICUs lack multidisciplinary teams or protocols for pediatric airway management, leading to suboptimal care for Post-Extubation Laryngitis (PEL). This results in failures to diagnose at-risk children or identify those who may develop airway obstruction post-discharge.

There is also an insufficient understanding of PEL pathophysiology and evolution into untreated airway obstruction even weeks after intubation.^26^ Not recognizing PEL symptoms and progression leads to discharging stridorous patients without follow-up. Unfortunately, these patients often return to emergency services weeks later in respiratory distress with established laryngeal stenosis, now facing risks of difficult intubation.

One-third of children awaited definite diagnoses or surgeries after hospital discharge due to long waiting lists for procedures requiring general anesthesia. Strategies to reduce these waiting lists are needed, although patients' comorbidities may also limit definitive treatment and decannulation.

Limitations include the retrospective nature of this study. Also, the small sample size and 23.68% loss to follow-up limit outcome assessment. Prospective studies and better strategies to track discharged PICU patients could help understand post-intubation needs and burdens. This includes monitoring possible ongoing laryngeal stenosis after PEL, not just in tracheostomy patients but also to allow better follow-ups for all.

We emphasize the importance of implementing otolaryngological evaluation and follow-up protocols and expanding access to therapeutic resources required to manage these children adequately.

## Conclusion

Post-extubation laryngitis is commonly confirmed upon suspicion. The severity of laryngeal lesions was linked to poorer one-year outcomes. Although NPL is a useful diagnostic tool, the need for examination under general anesthesia through MLB is high. A third of patients needed a tracheostomy and a fifth developed laryngeal stenosis. Otolaryngological evaluation, follow-up, and therapy access are crucial for managing intubation sequelae in children.

## Funding

The authors have no funding or financial relationships.

## Conflicts of interest

The authors declare no conflicts of interest.
